# Expansion of Thailand’s Social Pension Policy and Its Implications for Family Support for Older Persons

**DOI:** 10.1093/geroni/igaa057.1107

**Published:** 2020-12-16

**Authors:** Bussarawan Teerawichitchainan

**Affiliations:** National University of Singapore, SINGAPORE, Singapore

## Abstract

Thailand is among few developing countries that have provided universal social pension for its older adults since 2009. Analyzing nationally-representative data from the Surveys of Older Persons in Thailand, we address the extent to which older Thais have benefited from the policy and describe the socio-demographic correlates of older persons who primarily rely on OAA as their main income source. Importantly, we examine how reliance on OAA may have implications for intergenerational exchanges between older parents and adult children as well as the well-being of older Thais. Results show significant changes over the last decade in the patterns of old-age income sources and filial economic support for older parents, particularly after the universalization of the OAA policy. We find the declining importance of children and rising significance of OAA as the primary income source. Older persons whose main income source was OAA were considered socially and economically vulnerable, although it was men rather than women who were more likely to depend on OAA as the main income source. Results further indicate that reliance of OAA as the main income source is not associated with a reduction in non-monetary familial support (intergenerational coresidence and social support) for aged parents. Nevertheless, we find that older parents whose main income was OAA were consistently less likely to report income adequacy and psychological well-being compared to others. This evidence suggests that while the OAA scheme may have tackled old-age poverty issues, it has little impact in dampening economic and social inequality among older Thais.

